# Dissecting the Roles of the Autonomic Nervous System and Physical Activity on Circadian Heart Rate Fluctuations in Mice

**DOI:** 10.3389/fphys.2021.692247

**Published:** 2021-10-18

**Authors:** Nour Barazi, Nazari Polidovitch, Ryan Debi, Simona Yakobov, Robert Lakin, Peter H. Backx

**Affiliations:** Department of Biology, York University, Toronto, ON, Canada

**Keywords:** heart rate, autonomic nervous system, physical activity, mice, circadian rhythm

## Abstract

Heart rate (HR) and blood pressure as well as adverse cardiovascular events show clear circadian patterns, which are linked to interdependent daily variations in physical activity and cardiac autonomic nerve system (ANS) activity. We set out to assess the relative contributions of the ANS (alone) and physical activity to circadian HR fluctuations. To do so, we measured HR (beats per minute, bpm) in mice that were either immobilized using isoflurane anesthesia or free-moving. Nonlinear fits of HR data to sine functions revealed that anesthetized mice display brisk circadian HR fluctuations with amplitudes of 47.1±7.4bpm with the highest HRs in middle of the dark (active) period (ZT 18: 589±46bpm) and lowest HRs in the middle of the light (rest) period (ZT 6: 497±54bpm). The circadian HR fluctuations were reduced by ~70% following blockade of cardiac parasympathetic nervous activity (PNA) with atropine while declining by <15% following cardiac sympathetic nerve activity (SNA) blockade with propranolol. Small HR fluctuation amplitudes (11.6±5.9bpm) remained after complete cardiac ANS blockade. Remarkably, circadian HR fluctuation amplitudes in freely moving, telemetrized mice were only ~32% larger than in anesthetized mice. However, after gaining access to running wheels for 1week, circadian HR fluctuations increase to 102.9±12.1bpm and this is linked directly to increased O_2_ consumption during running. We conclude that, independent of physical activity, the ANS is a major determinant of circadian HR variations with PNA playing a dominant role compared to SNA. The effects of physical activity to the daily HR variations are remarkably small unless mice get access to running wheels.

## Introduction

Almost every physiological system in the body possesses a circadian rhythm which is modulated by the superchiasmatic nucleus (SCN), the master clock of the mammalian brain (Foster, 1998). Circadian variations in the cardiovascular system (CVS) are especially apparent with marked daily fluctuations in heart rate (HR) and blood pressure (Zhang and Sannajust, 2000; Dilaveris et al., 2001). Circadian variations in the CVS are particularly important because the risk of adverse events, such as myocardial infarction, ischemia, atrial arrhythmia, stroke, and sudden cardiac death peak prominently in the early morning hours (Guo and Stein, 2003; Alibhai et al., 2015). Indeed, the timing of the adverse CVS events is strongly associated with increases in HR and blood pressure in anticipation of waking and activity (Coats et al., 1992; Shiotani et al., 2009), which have been linked to many factors including circulating catecholamines and the activity level of autonomic nerve system (ANS) (Guo and Stein, 2003; Scheer et al., 2009), with the parasympathetic nervous activity (PNA) showing a more dominant role than sympathetic nervous activity (SNA) (Burgess et al., 1997; Kalsbeek et al., 2006).

Although many studies have previously established that the ANS plays a role in circadian HR fluctuation in humans (Burgess et al., 1997; Massin et al., 2000; Boudreau et al., 2012) and other species (Warren et al., 1994; Kriegsfeld et al., 2004) including mice (Tong et al., 2013), recent studies have suggested that diurnal variation in the intrinsic beating rates of the sinoatrial (SA) node is the dominant factor in controlling diurnal HR fluctuation in mice (D'Souza et al., 2020), with the ANS playing a minor role (Yamashita et al., 2003; Durgan et al., 2005). This conclusion seems at odds with the expectation that physical activity causes HR changes (Xiang and Hester, 2016) by recruiting the ANS in order to help match cardiac output with tissue demands during exercise (Gordan et al., 2015; Xiang and Hester, 2016).

No previous studies have dissected the dependence and interdependence of the daily HR variations on cardiac ANS activity versus physical activity. Thus, we compared circadian HR fluctuations in (immobilized) anesthetized mice versus telemetry-implanted awake mice, before and after access to running wheels. We found that immobile anesthetized mice display ANS-dependent circadian HR fluctuations with amplitudes indistinguishable from conscious free-moving mice. Interestingly, only after gaining access to running wheels do daily HR variations increase in association with increased daily physical activity in mice.

## Materials and Methods

An expanded Methods section is available in the online data supplement. Ethical approval for all procedures and handling of mice during the student was reviewed and granted by the York University Animal Care Committee (ACC). All experimental protocols conformed to the standards of the Canadian Council on Animal Care.

### Experimental Animals

Male CD1 mice (6–8weeks, 32-39g) were purchased from the Charles River Laboratories (Montreal, QC, Canada). All mice were housed at the Vivarium, Department of Biology, York University, with 12-h light cycles corresponding to their respective conditions (light-dark or dark-light), as described in the expanded methods.

### Electrocardiographic Measurements in Anesthetized Mice

To assess circadian fluctuations in heart rate (HR), surface ECG (sECG) recordings were made in mice (*n*=13) that were anesthetized with a 1.5% isoflurane/oxygen mixture for periods lasting 1.5–2h. The sECG recordings were made in the lead II arrangement of sub-dermal platinum electrodes. While anesthetized, internal core body temperatures in mice were maintained between 36.9 and 37°C. Ponemah Physiology Platform (P3) software was used to estimate HR R-R intervals, averaged over 25-min periods.

The HR estimates were made at four different (ZT) time points: (1) the beginning of the light phase (ZT 0/24), with ZT 0 assumed to be equal to ZT 24 based on the 12h:12h light-dark cycle; (2) 6h into the light phase (ZT 6); (3) the onset of the dark cycle (ZT 12); and (4) 6h into the dark phase (ZT 18). Two groups of mice were used. In the first group, the mice (*n*=7) were initially housed in rooms either with regular light-dark (LD) cycles in order to estimate HRs at ZT0/24, ZT 6, and ZT 12 (*n*=4) or with reverse dark-light (DL) cycles in order to determine HRs at ZT 12, ZT 18, and ZT 24/0 (*n*=3). After estimating HR for each mouse on three separate occasions (with each recording separated by at least 2days), the mice in the LD rooms were moved to the rooms with DL lighting and vice versa. After waiting 10days, to allow mice to acclimatize to the new light cycle (data not shown), HRs were again determined in triplicate at the appropriate time points. In the second group, mice were housed in rooms either with LD cycles (*n*=3) or with DL light cycles (*n*=3). HRs were measured in triplicate at the appropriate times. Since detailed comparisons revealed no measureable differences in daily HR variations between the two groups of mice, the data from all 13 mice were pooled for all the analyses presented.

Each time mice were anesthetized, sECG recordings were made before and after administration of autonomic blockers. Autonomic blockers were introduced in two different sequences: (1) intraperitoneal (IP) injection of propranolol (10mg/kg BW) to block cardiac sympathetic nerve activity (SNA) followed by IP atropine (2mg/kg BW) injection to achieve total autonomic blockade or (2) IP atropine injection to block parasympathetic nerve activity (PNA) followed by propranolol IP treatment to (again) achieve total cardiac autonomic blockade. HRs were estimated from the sECG recordings 25min after the administration of each autonomic blocker.

### Telemetric Electrocardiography

Diurnal fluctuations in HR of conscious mice (*n*=5) were measured by surgically implanting radio telemetry surface electrocardiogram (ECG) units in lead II arrangement (F-E7, Grass Technologies, West Warwick, RI, United States). Following 1week of post-operative recovery, 48- or 72-h recordings were collected in the absence of a running wheel (week 1) and subsequently 7days after the introduction of a running wheel (week 2). All data were analyzed using the Ponemah Physiology Platform (P3) software. HRs were derived for each time point from R-R intervals averaged over 120min/data point.

### Respirometry Measurements

Mice were housed individually in custom-built metabolic cages. Mice were housed in and recorded from in metabolic cages for 48 (Monday to Wednesday or Wednesday to Friday) or 72h (Friday to Monday), with hours 24–48 analyzed to ensure consistency between recording conditions. Humidified (~40% H_2_O) air was continuously injected into the metabolic cages at 1l/min. Simultaneous tracking of running parameters (running speed and cumulative running distance) was performed using custom-built running wheels and atmospheric parameters (air temperature, atmospheric pressure, and %H_2_O) using BME-280 sensors (Adafruit, New York City, United States). Outgoing cage gas (%O_2_) was desiccated and sampled using an iWORX GA-200 O_2_/CO_2_ gas analyzer (iWORX Dover, United States) at 0.5l/min. V_O2_ consumption rates were calculated, using a standard respirometry equation:


$$V_{\mathrm{O2}}=\Delta \%O_{2}*\left(P_{\mathrm{atm}}-P_{H2O}\right)*\;F$$

where ∆%O_2_ = difference in %O_2_ between incoming and outgoing air flowing, F is the rate of air flow into the cage, P_atm_ = atmospheric pressure, and P_H2O_ = partial pressure of H_2_O = f (%humidity and temperature).

### Statistical Analysis

HR data results are reported as mean±standard deviation (SD). To detect circadian HR fluctuations, the pooled HR data for all the mice, at different time points, were fit nonlinearly using the Least Squares (ordinary) Fit algorithm, provided through the program GraphPad Prism (GraphPad Software, Inc.) to the following sine function:


$$Y=A*\;\sin\;\left(\left(2*\;\pi *\;T/24\right)+\phi \right)+B$$

where A is the amplitude (i.e., one half the peak to trough variation) expressed in heart beats per minute, ϕ is the acrophase (i.e., the time delay from ZT0 of the HR cycle) expressed in radians, B is the baseline offset which equals the mean heart rate over 24h (i.e., the MESOR) expressed in heart beats per minute, and T is the time of day in hours, π=Pi radians=3.14159 radians. Fits were constrained to a 24-h wavelength given the controlled 12:12 light-dark cycle. Zero amplitude (no rhythm) tests were used to determine the presence of a circadian rhythm in HR. Differences in fit parameters were determined within and between groups using an extra sum-of-squares F test. To assess the robustness of our approach, the presence of circadian HR fluctuations was also assessed using nonlinear fits the HR results for each individual mouse to a sine function (Supplementary Figure 1). In this case, the nonlinear fitting routine provided an estimate of “A” and “ϕ” for each mouse which was then used to determine whether “A” (mean ± SD) was non-zero using a T-Test (*p* < 0.05). A two-way mixed-model repeated measures ANOVA with Holm- Šidak correction for multiple comparisons was used to compare circadian HR fluctuations between groups of anesthetized (i.e., with and without pharmacological blockade) or conscious mice (i.e., with or without free wheel access). An independent (two tailed) student’s t-test was used to assess differences between anesthetized and conscious mice. Values of *p* < 0.05 were considered significant.

## Results

### Circadian Rhythm in Anesthetized Mice

**Figure 1:**
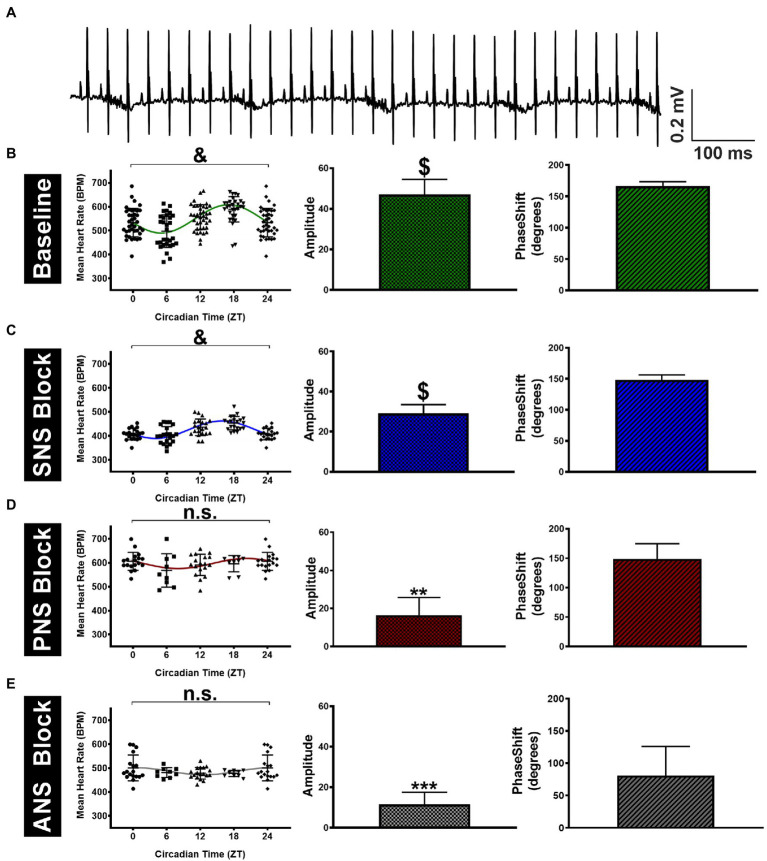
Variation in surface ECG recorded mean HR of anesthetized mice throughout the day. **(A)** Sample telemetry ECG tracing recorded at ZT 12 at a sampling rate of 5kHz. **(B)** The left panel shows the estimated HRs across four time points (ZT0 data are the same as ZT24) for mice (*n*=13) under baseline conditions. The green line represents the sine function that yields the best nonlinear fit to the HR data shown. The non-zero (*p* < 0.0001) amplitudes (middle panel) and the accompanying phase shifts (right panel) estimated from the best fits are shown. **(C)** Results are shown after SNA blockade with propranolol (10mg/kgi.p.) with significant (*p* < 0.0001) fluctuations in HR throughout the circadian day (left panel) accompanied by non-zero amplitudes in HR fluctuations (middle panel, *p* < 0.0001). **(D)** HR data after PNA blockade with atropine (2mg/kgi.p.) showed no significant fluctuations in HR (left panel, *p*=0.24) accompanied by non-zero amplitudes in HR fluctuations (middle panel, *p*=0.002). **(E)** HR data under total autonomic blockade with atropine + propranolol showing insignificant fluctuations in HR (**left panel**, *p*=0.31) contrasted by non-zero amplitudes in HR fluctuations (**middle panel**, *p*=0.0007). Data are presented as Mean±S.D. ^&^*p* < 0.0001 and n.s (*p*=0.24 for **D** and *p*=0.31 for **E**) using a mixed-model repeated measures ANOVA (left panel). ^**^p < 0.01, ^***^p < 0.001, and $*p* < 0.0001 based on the zero amplitude (no rhythm) test from nonlinear fits (middle panel).

We began by assessing the relative contribution of the autonomic nervous system (ANS) alone to circadian heart rate (HR) fluctuations in anesthetized mice to eliminate the contribution of physical activity to our measures. Triplicate surface electrocardiogram (sECG) recordings were made at four different time points (ZT0/24, 6, 12, and 18) to estimate HR, both before and after administration of autonomic blockers, with the repeated measurements always being separated by at least 2days (See Methods). HRs estimated from sECG recordings prior to administration of autonomic blockers (i.e., baseline HR) are shown in Figure 1 and Table 1. Despite considerable HR variability at each time point, mean HRs in anesthetized mice differed (*p* < 0.0001) over 24h. HRs at ZT18 (589±47bpm) were higher (*p*=0.019) and HRs at ZT 6 (498±55bpm) were lower (*p*= 0.002) than the mean HRs (544±65bpm) averaged over all four time points (Figure 1B; Table 1). To quantify circadian HR fluctuations, we next performed nonlinear fits of the HR data to sine functions with 24-h periods. These fits yielded non-zero (*p* < 0.0001) sine function amplitudes of 47.1±7.4bpm with phases of 2.91±0.12 radians (11.1±0.5h). These fits demonstrate the ZT 6 and ZT 18 align closely with the minimum and the maximum, respectively, of the sinusoidal pattern of HR variation, similar to previous findings in conscious mice (Arraj and Lemmer, 2006).

**Table 1:** Summary of amplitudes and phases estimated from nonlinear sine function fits to the HR measurements under the indicated experimental conditions.

Condition	*N*	Absolute HR			Normalized HR	
		Mean HR (BPM)	Amplitude	Phase (radians)	Amplitude	Phase (radians)
**Anesthetized Mice**						
Baseline	13	544±65	47.1±7.4	2.91±0.12	0.09±0.02	2.91±0.17
SNA Block	7	424±36^***^	29.1±4.4^*^	2.59±0.14	0.07±0.01^n.s^	2.64±0.19
PNA Block	6	592±46^***^	16.4±9.4^*^	2.59±0.46	0.02±0.02^*^	2.43±0.68
ANS Block	6	485±36^***^	11.9±5.9^**^	1.41±0.79	0.03±0.01^**^	1.42±0.77
**Conscious Mice**						
SED	5	568±54	65.9±13.3	2.84±0.18	0.12±0.03^*^	2.84±0.24
WHEEL	5	577±91	122.5±11.6^**^	2.45±0.09	0.18±0.03^*^	2.33±0.17

The amplitudes and phase shifts were obtained by nonlinear fits of the HR results to sine functions for various conditions: Baseline = no blockers, SNA Block = with propranolol, PNA Block = with atropine, ANS Block = with atropine and propranolol, SED = conscious mice with no wheel access, and WHEEL = conscious mice with wheel access. Data presented as Mean ± S.D.

* *p*<0.05

** *p*<0.01

*** *p*<0.001, and ^n.s^(P=0.06) compared to baseline or sedentary mice with no wheel access using an extra sum-of-squares F test. Additional within- and between-group comparisons are outlined in the Results.

In assessing the contribution of the cardiac ANS to circadian HR fluctuations, SNA blockade (alone) caused the mean HRs (averaged over all time points) to decrease (*p* < 0.0001) to 424±36bpm compared to HRs prior to propranolol treatment (Figure 1C; Table 1), consistent with a high basal cardiac SNA in mice (Gehrmann et al., 2000). Despite these HR reductions, circadian HR fluctuations remained after SNA blockade as demonstrated by the presence of non-zero (*p* < 0.0005) sine wave amplitudes of 29.1±4.4bpm but these amplitudes were reduced (*p*=0.045) compared to the mice before SNA blockade. Moreover, the estimated phase of the sine functions was not different (*p*=0.22) between mice after propranolol treatment (2.59±0.14 radians; 9.9±0.5h) compared to baseline. By contrast, when PNA was blocked with atropine, the HRs averaged over all time points were elevated (*p* < 0.0001) to 593±46bpm, compared to baseline (Figure 1D; Table 1), as reported previously (Lakin et al., 2018). Additionally, after PNA blockade, the amplitudes of sine functions were non-zero (*p*=0.007) and were reduced (*p*=0.030) to 16.4±9.4bpm compared with baseline. However, the phase of the circadian fluctuations after PNA blockade (2.59±0.46 radians; 9.9±1.8h) was again not different (*p*=0.16) from phases measured under baseline conditions (or following SNA blockade). Although previous studies concluded that ANS blockade eliminates circadian HR fluctuations (Tong et al., 2013), the amplitudes in anesthetized mice remained non-zero (*p*=0.007) after treatment with both atropine and propranolol (11.9±5.9bpm), although there was a trend (*p*=0.12) in the phase being shifted relative to untreated anesthetized mice to 1.41±0.79 radians (5.4±3.0h; Figure 1D; Table 1). It is worth mentioning that we also performed separate nonlinear fits of the HR results for each mouse as summarized in Supplementary Figure 1 and Supplementary Table 1. Analyses of the results using individual HR fits lead to the same conclusions made using global fits (Table 1), which demonstrates the robustness of the circadian HR fluctuations in anesthetized mice.

The results presented thus far suggest that PNA influences daily HR variations much more than SNA. However, because HRs were affected differently by PNA versus SNA blockade, we also normalized circadian HR amplitudes by the mean HRs for each condition. As summarized in Table 1 (rightmost columns), these analyses revealed that the relative circadian HR fluctuations were reduced by almost ~75% by PNA blockade (i.e., 0.09 at baseline versus 0.02 after atropine) while being reduced by only ~16% by SNA blockade (i.e., 0.09 to 0.07). Moreover, the relative daily HR fluctuations after complete ANS blockade were comparable (*p*=0.68) to the relative fluctuation seen with PNA blockade alone. Similar findings and conclusions were made when using normalized HR in analyses using nonlinear fits to individual mice (Supplementary Table 1, rightmost columns). These results establish that PNA plays a dominant role in the circadian HR fluctuations in anesthetized mice compared to SNA.

### HR Measurements in Conscious Mice

**Figure 2:**
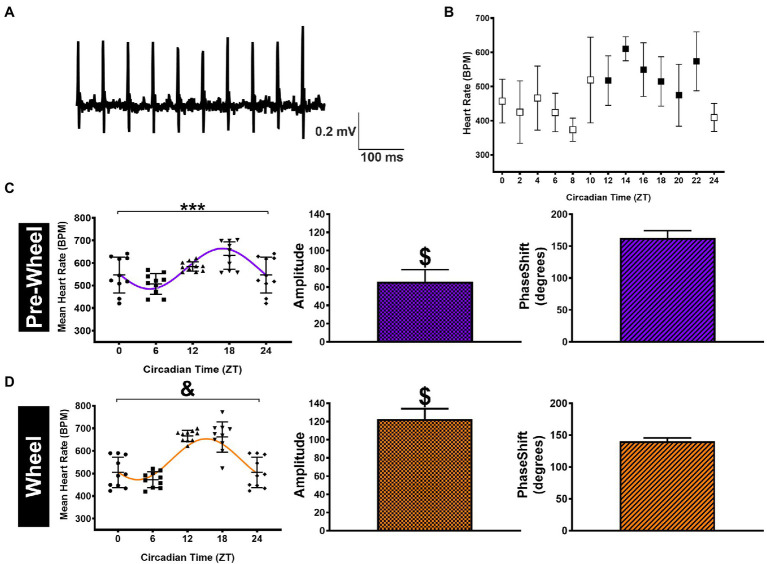
HR measurements and analyses in telemetrized conscious mice. **(A)** Representative sample telemetry ECG tracings recorded from a sedentary mouse at ZT 12. **(B)** HR data (*n*=5 mice) displayed in 2-h intervals with the mean HR determined by averaging the HRs estimated for 1h before and 1h after the time point shown (open symbols = light phase and filled symbols = dark phase). **(C)** HR data for conscious (pre-wheel) mice at ZT 0, ZT 6, ZT 12, and ZT 18 (ZT0 = ZT24)(left panel). The line shows the curve predicted by nonlinear sine function fits to the HR data with amplitude (middle) and phase (right) estimated from the nonlinear fits shown as Mean ± SD. **(D)** The same data as in **(C)** but for conscious mice after having access to running wheels for 1week. ^***^p < 0.001 and ^&^*p* < 0.0001 using a mixed-model repeated measure (left panel). ^$^*p* < 0.0001 based on the zero amplitude (no rhythm) test from nonlinear fits (middle panel).

The presence of circadian HR fluctuations in anesthetized mice was somewhat unexpected because daily HR fluctuations as well as changes in cardiac ANS modulation are linked strongly to changes in physical activity (Kalsbeek et al., 2006). Therefore, to determine how physical activity might also contribute to circadian HR fluctuations, we continuously measured HRs over a 24-h period in conscious free-moving mice (*n*=5) with implanted ECG telemetry devices. Specifically, mean HRs were estimated every 2h over a 24-h period by averaging HRs for 1hour before and 1hour after each time point as displayed in Figure 2B. Although the mean HRs in conscious mice (averaged over a 24-h period) were not different (*p*=0.12) from anesthetized mice (i.e., 568±54bpm versus 544±66bpm), the non-zero (*p* < 0.0001) sine function amplitudes, estimated from nonlinear fits using HRs at ZT 0/24, ZT 6, ZT 12, and ZT 18 to match anesthetized mice, were higher (*p*=0.0007) than in anesthetized mice (i.e., 65.9±13.3bpm). Similar estimates of the circadian HR amplitudes (69.2±3.3bpm) and phase (2.84±0.12 0.18 radians; 10.9±0.7h) were observed when nonlinear fits were performed using all HRs (i.e., 12 time points) over a 24-h period. As expected, the normalized HRs in the conscious mice were also greater (*p*=0.009) than anesthetized mice (0.12±0.03 in conscious mice versus 0.09±0.02 for baseline) without differences (*p*=0.89) in phase (Table 1). These results in conscious mice are similar to previous studies (Arraj and Lemmer, 2006), consistent with activity contributing to circadian HR fluctuations.

**Figure 3:**
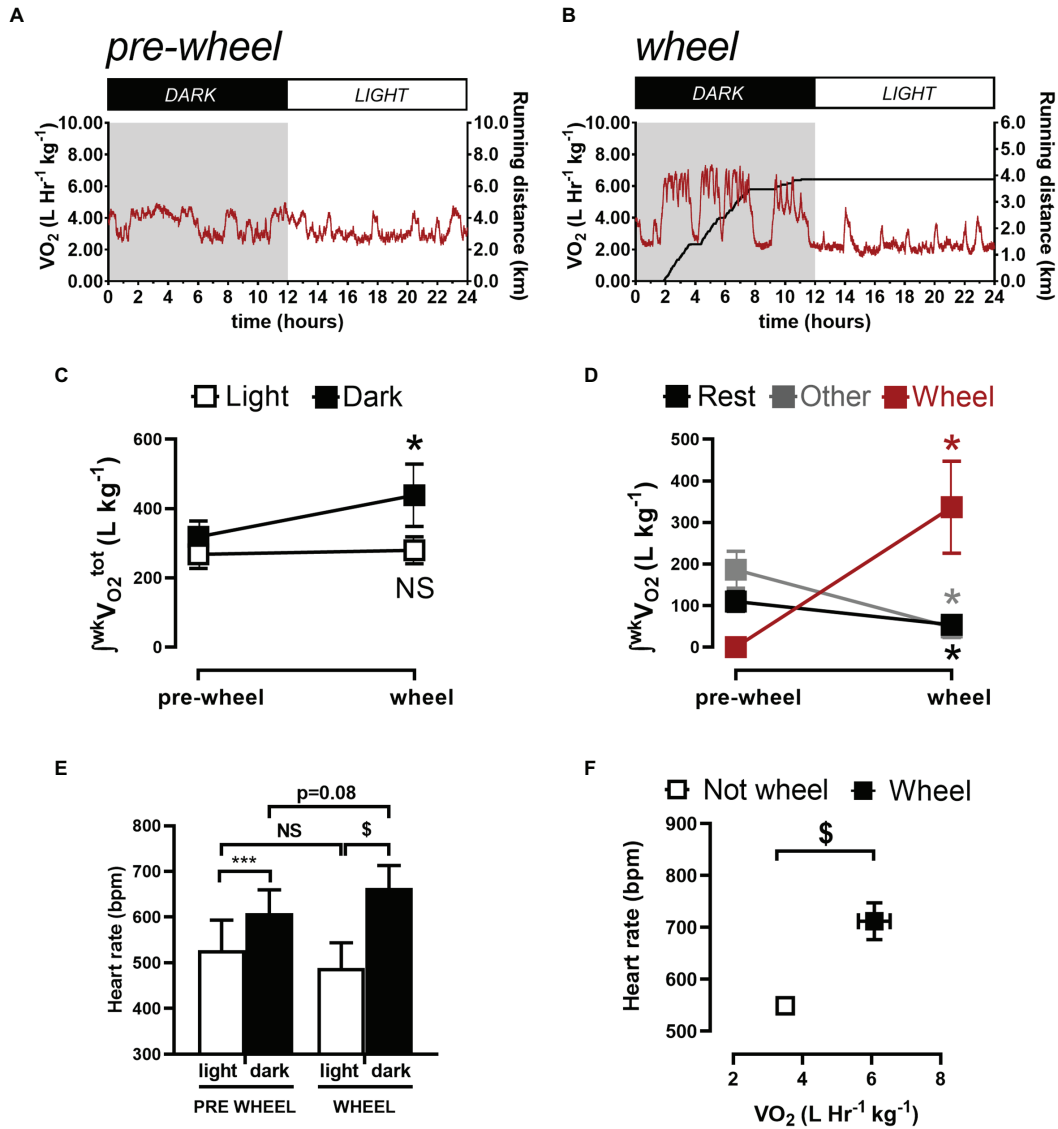
Quantification of weekly total oxygen consumption (∫^wk^V_O2_) associated with rest, non-rest/non-wheel (other) activity, and running (wheel) activity. Sample 24-h V_O2_ recordings before **(A)** and 1week after **(B)** a CD1 mouse gained access to a running wheel. **(C)** shows the integrated O_2_ consumed per week by mice during the light (sleeping) phase and during the dark (active) phase, before and after gaining wheel. **(D)** shows the integrated O_2_ consumed per week during the dark phase quantified during rest (sleeping), with wheel running and with “other” activities (i.e., neither sleeping or wheel running). **(E)** summarizes the HR results during the light and dark phases from before and after gaining access to running wheels. The data reveals that the HR differed between the light and dark phase for mice before and after gaining access to running wheels. HRs in the dark phase increased (*p*=0.02) while trending (*p*=0.08) toward reductions during the light phase after mice gained access to running wheels for 1week. **(F)** shows the relationship between HR and the V_O2_ when mice are not on the wheel versus when they are on the running wheel. Data are presented as Mean ± SD. ^*^p < 0.05, ^***^p < 0.001, and ^$^p < 0.0001 using a repeated measures one-way ANOVA with Holm-Šidak *post-hoc* test.

To further assess whether the differences in circadian HR fluctuations between conscious and anesthetized mice were related to physical activity, O_2_ consumption rates (V_O2_) were measured in conscious mice and typical measurements are illustrated in Figure 3A. Such recordings revealed (Figures 3C) that the amount of O_2_ consumed (i.e., integrated V_O2_) during dark (active) periods (319±19l/kg/week) was higher (*p*=0.008) than during the sleep period (267±17l/kg/week) which correlated with higher HRs (609±51bpm) during the dark phase versus during the light phase (527±66bpm) as summarized in Figures 3E.

Although these observations suggest that the larger circadian HR variations in conscious mice compared to anesthetized mice arise from differences in physical activity, previous studies have established that anesthesia can alter ANS regulation of heart function and HR (Janssen et al., 2004; Constantinides et al., 2011). To explore these links between physical activity and circadian HR variations, we analyzed the relationships between HRs and O_2_ consumption in conscious mice (*n*=5) after gaining access to running wheels because access to running wheels increases physical activity in mice (Yasumoto et al., 2015). We focused our analyses on mice after having running wheels access for 1week to minimize the potential confounding effects of physical conditioning on basal HR and ANS remodeling (Aschar-Sobbi et al., 2015; Billman et al., 2015; Lakin et al., 2018), while at the same time allowing mice to become familiarized with using the running wheel. Consistent with previous studies (Yasumoto et al., 2015; Kolbe et al., 2019), we found that after 1week mice ran 4.0±2.5km/day, exclusively during the dark phase which was associated with increased (*p*=0.010) circadian HR fluctuations to 122.5±11.6bpm and a trend (*p*=0.083) toward a shift in phase (2.45±0.12 0.09 radians; 9.36±0.12 0.3h; Figure 2D; Table 1), as estimated with nonlinear sine fits to HR data measured at ZT0/6/12/18. Moreover, these changes in circadian HR fluctuations following wheel access were associated with increases (*p*=0.01) in the total (integrated) O_2_ consumption (to 438±38l/Kg/week) as well as increases (*p* < 0.0001) in HR (to 664±49bpm) during the dark, without notable changes in O_2_ consumption (*p*=0.53) or HR (*p*=0.082) during the light (rest) phase, compared to before wheel access (Figures 3C,E). Thus, changes in O_2_ consumption align with changes in HR.

To more directly demonstrate connections between HR changes and physical activity, we subdivided the O_2_ consumption rates into three different categories of activity: “resting/sleeping,” wheel running, or “other” (See Methods). The identification of different categories can be readily appreciated by examining Supplementary Figure 2 (see also Figures 3A,B), which display two discernable peaks in the V_O2_ histograms before wheel access versus 3 peaks when wheels are present with highest peak in the V_O2_ histogram aligning with wheel running during the dark phase (Figure 3B). Moreover, these analyses clearly reveal that the increased O_2_ consumption during the dark phase (Figure 3C) is associated with wheel running, because of large drops (*p* < 0.05) in O_2_ consumption after wheel introduction during “resting” (from 24±10 to 18±7l/Kg/week) as well as during “other” activities (209±31 to 48±24l/Kg/week), as summarized in Figure 3D. More important, running HRs were 664±49bpm which are far greater (*p* < 0.0001) than the HRs measured when mice are not running (489±55bpm) and these HR differences correlated directly with corresponding differences in mean V_O2_ (Figure 3F). Thus, we can conclude that, while ANS strongly influences circadian HR fluctuations independent of physical activity, the circadian HR variations can also be strongly influenced by daily alterations in physical activity.

## Discussion

Our study is the first to compare circadian HR oscillations in immobilized (anesthetized) mice with conscious free-moving mice to assess the relative contributions of the ANS (alone) and physical activity to circadian HR fluctuations in mice. We found that anesthetized mice display prominent daily HR variations characterized by troughs at ZT 6 (middle of the sleep phase) and peaks at ZT 18 (middle of the active phase). Nonlinear fits of the HR results to sine functions (with a 24-h period) revealed that circadian fluctuations in HR occur in anesthetized immobilized mice with amplitudes of ~12% of the mean HR. Moreover, the amplitude of circadian HR fluctuations in anesthetized mice was reduced by ~16% with sympathetic blockade and by ~75% following parasympathetic blockade. Thus, the sympathetic and parasympathetic arms of the ANS both contribute to circadian HR fluctuations with PNA having an ~5-fold greater influence on circadian HR variations in anesthetized mice. These conclusions align well with previous studies in humans demonstrating a far stronger connection between PNA and the circadian fluctuations of HR in humans (Burgess et al., 1997; Scheer et al., 2004). This similarity in circadian HR regulation between mice and humans seems especially germane given that basal HR regulation in mice is dominated by high SNA (Ishii et al., 1996; Gehrmann et al., 2000; Lakin et al., 2018) while the opposite is seen in humans (Krum et al., 1991; Burgess et al., 1997; Scheer et al., 2004).

Although the basis for the discordant ability of PNA to regulate baseline HR versus circadian HR variations is unclear, these observations are consistent with previous studies showing that increased SNA induced by physiological stress can entrain peripheral clocks without directly influencing the master circadian hypothalamic regulator in the suprachiasmatic nucleus (SCN) (Scheer et al., 2003; Tahara et al., 2017). Indeed, the SCN, which integrates numerous inputs with daily photic fluctuations, directly entrains hypothalamic paraventricular nuclei as well as the cardiac centers in the brain stem (Benarroch, 1993; Silvani et al., 2016), which together control the ANS balance (Scheer et al., 2004; Shea et al., 2011) as well as hormonal secretion (Boudreau et al., 2012; Morris et al., 2012) in anticipation of changes in physical and mental activities. It seems reasonable to suggest that daily variations in ANS activity originating from the cardiac center are important, or possibly the dominant, determinants of the circadian HR fluctuations seen in our anesthetized mice. Clearly additional studies involving direct measurements of the cardiac ANS will be needed to test this assertion.

Our studies also revealed that following complete autonomic blockade circadian HR variations remained, which is consistent with previous studies in humans (Guo and Stein, 2003; Boudreau et al., 2012) and rodents (Buijs et al., 2003; Engeland and Arnhold, 2005; Schroeder et al., 2011; Tong et al., 2013). Moreover, given that the amplitudes of the HR fluctuations after complete ANS blockade are similar to those seen following PNA blockade alone, and given that SNA blockade also reduced daily HR variations, our results demonstrate a non-additivity of the PNA versus SNA in circadian HR regulation. This is not surprising since blockade of one arm of the ANS will impact *via* various feedback on the activity of the other arm (i.e., accentuated antagonism) (Levy, 1971). Regardless, although we did not test the underlying basis for the residual HR variations when the cardiac ANS was blocked, previous studies have established that circulating humoral factors, such as catecholamines (Scheer et al., 2010), cortisol, and the Renin-Angiotensin-Aldosterone System (RAAS; Zucker et al., 2014), vary in a circadian manner and can directly influence the SA node (Yee et al., 2001; Chung et al., 2011). The circadian variations of these humoral factors appear to be under the control of the SCN which uses vasoactive intestinal peptide (VIP) to directly and indirectly modulate the release of adrenocorticotropic hormone (ACTH; Buijs et al., 2003; Boudreau et al., 2012; Morris et al., 2012). On the other hand, previous studies have concluded that circadian HR fluctuations arise from daily changes in the intrinsic properties of the SA node (i.e., local circadian cardiac clock; Wang et al., 2021), arising from changes in the expression of HCN4-based pace-maker channels (Oosting et al., 1997; Ptaszynski et al., 2018; D'Souza et al., 2020) or other channels including Kv4.2 (Yamashita et al., 2003) and Kv1.5 (Durgan et al., 2005). While these variations can clearly contribute to the daily HR variations seen in the absence of ANS activity, our studies establish that the ANS plays a necessary and dominant role in controlling basal circadian daily HR fluctuations in anesthetized mice. Importantly, this control can arise either from variations in ANS activity or from diurnal variations in the responsiveness of the SA nodal cells to the cardiac PNA and SNA (Buijs et al., 2013) or from combinations of these factors. Clearly, additional studies, particularly direct measures of cardiac ANS activity, will be needed to distinguish between these possibilities.

As expected, circadian HR fluctuations were readily observed in conscious, free-moving, and telemetry-implanted mice, as reported previously in rodents (Arraj and Lemmer, 2006; Tong et al., 2013). Since HR is tightly linked to physical activity *via* altered cardiac ANS (Dishman et al., 2006) and anesthesia is known to alter ANS regulation of HR and cardiac function (Janssen et al., 2004; Constantinides et al., 2011), it was somewhat unexpected that the magnitude of the circadian HR fluctuations was similar between free-moving conscious mice and immobilized anesthetized mice. Indeed, isoflurane can profoundly alter both sympathetic (Seagard et al., 1984; Kato et al., 1992) and parasympathetic (Picker et al., 2001) cardiac ANS control given its sympatholytic and vagolytic effects, which would be expected to attenuate ANS-mediated circadian HR fluctuations. However, the lack of significant differences between groups in the current study might be related, at least in part, to the relatively small changes in HR associated with increased ambulatory activity in mice (Lujan and DiCarlo, 2013), at least compared to humans. Nonetheless, in the absence of running wheels, the total oxygen consumed by mice during their active (dark) phase was also only marginally greater (i.e., ~16%) than during their inactive (light) phase, consistent with previous studies (Aschoff et al., 1973; Zhang and Sannajust, 2000; Pfeffer et al., 2015; Yasumoto et al., 2015). Ultimately, any differences in HR and O_2_ consumption between the dark and light phase will depend on the relative physical activity during the two phases. In this regard, the contributions of physical activity to circadian HR fluctuations became far more apparent after mice have access to running wheels and this is clearly a result of a strong entrainment of physical activity associated with wheel running during the dark phase which increases O_2_ consumption. Specifically, after gaining access to running wheel, circadian HR fluctuations nearly doubled and this was associated with a nearly ~40% increase in total O_2_ consumption in the dark (active) phase which is directly linked to increases in V_O2_ when mice are wheel running. These findings are similar to previous studies in mice (Yasumoto et al., 2015) as well as rats (Seifert and Mortola, 2002). Moreover, it was also apparent that added O_2_ consumption linked to wheel running occurred in conjunction with reduced O_2_ consumption associated with other activities (i.e., resting and “other”) during the dark phase which inherently required lower V_O2_. An additional interesting, and less obvious, consequence of wheel running was a reduction in mean HRs during the light phase which was associated with small reductions in O_2_ consumption. The basis for this reduction in HR in the light phase is unclear but may arise from the known strong impact of exercise (increased physical activity) on SCN entrainment (Edgar and Dement, 1991; Schroeder et al., 2012), neuronal activity (Yamazaki et al., 1998; Schaap and Meijer, 2001), and on other factors, such as body temperature (Yasumoto et al., 2015) and plasma corticosteroid levels (Droste et al., 2003). Alternatively, the minimal reductions in O_2_ consumption might reflect the need for muscle rebuilding during sleep as previously shown (Vanuxem et al., 1997; Yang et al., 2019). However, the explanation may be simpler, as the lower O_2_ consumption during the light phase may reflect a shift in behaviors, with increased dark phase running yielding to rest or less frequent and/or less intense activity during the light cycle.

Unlike the amplitude, the phase of circadian HR oscillations was similar between all the groups except for an apparent trend toward a phase shift following complete autonomic blockade. Interestingly, we did not see any phase shifts in our anesthetized mice even though anesthesia for prolonged periods (>3h) can shift circadian rhythms in a time-dependent manner following recovery by varying extents depending on the depth, timing, and duration of the anesthesia (Kikuchi et al., 2013) associated with changes in the expression of clock genes in the SCN (Orts-Sebastian et al., 2019) and the periphery (Xia et al., 2015). In our studies, no significant phase shifts were observed over time in our mice, despite multiple exposures (typically nine times over a 3-week period) at various times of the day (i.e., different time points). The absence of phase shifts in our mice may reflect the relatively short durations (<1.5h) and low concentrations (1.5%) of isoflurane administration or re-synchronization with the light cycle in between measurements. The similarity in the phase shifts in the circadian HR variations following acute blockade of the either the PNA or SNA alone is consistent with previous studies showing that the levels of cardiac PNA and SNA are regulated in a coordinated and reciprocal manner by both the dorsal motor nucleus of the vagus nerve and the nucleus ambiguous as well as the rostral ventrolateral medulla in the medullary cardiac center (Benarroch, 1993). On the other hand, we found that complete ANS blockade was associated with a trend toward phase shifts in the circadian HR fluctuations. While the reliability of this conclusion can be questioned because of difficulties in estimating phases when sine wave amplitudes are small (as seen with complete ANS blockade), these observations are consistent with studies demonstrating advancement in the phase of the SA node transcriptome as well as humoral factors affecting HR relative to ANS activity (Martino et al., 2004; Tong et al., 2013), possibly in anticipation of daily HR increases (Wang et al., 2021). However, other studies found relatively good alignment between local cardiac clocks in the SA node and ANS activity (Scheer et al., 2003; Engeland and Arnhold, 2005). Clearly, additional studies are needed to assess the phase relationships between the ANS and other factors effecting daily HR variation. Finally, the enhanced circadian HR fluctuations after mice were presented with running wheels were not associated with significant phase shifts. This entrainment of the daily HR variation by exercise is consistent with previous studies showing that exercise can accelerate re-synchronization of circadian rhythms following disruption of light cycles in humans and animal models (Reebs and Mrosovsky, 1989; Mistlberger and Skene, 2004; Tahara and Shibata, 2018).

## Limitations

The influence of cardiac ANS on diurnal HR fluctuations was assessed indirectly with pharmacological blockade, with atropine and propranolol. However, these agents can profoundly alter the tonic activity of the sympathetic (Seagard et al., 1984; Kato et al., 1992) and parasympathetic (Picker et al., 2001) arms of the cardiac ANS control, in addition to depressing baroreflex control of HR (Lee et al., 2002).

It is conceivable that the HR changes seen with pharmacological blockade arose, at least in part, from off-target effects on selected ion channels in the SA node, such as Nav1.5 (Wang et al., 2010) or HCN4 (Tamura et al., 2009).

We did not directly measure autonomic nerve activity and it is conceivable that the ANS-dependent circadian HR fluctuations are dependent on intrinsic circadian changes of molecular factors that affect SA node function (Young et al., 2001; Yamashita et al., 2003; Durgan et al., 2007; D'Souza et al., 2020; Wang et al., 2021). Although we were able to detect circadian HR fluctuations in all the groups of mice studied using nonlinear fits of HR data to a single sine function with a 24-h cycle length, the inclusion of higher harmonics of the 24-h cycle would have likely provided a more robust fits and more accurate estimates of the amplitude of the circadian changes in HR.

## Conclusion

Our study establishes that brisk circadian HR rhythms are present in immobilized anesthetized mice, with PNA playing a (~5-fold) larger influence, than SNA, on these rhythms. Moreover, the amplitudes of the circadian HR fluctuations in conscious mice were remarkably similar anesthetized mice until mice gained access to running wheels whereupon these amplitudes increased by ~80% in association with increased physical activity and O_2_ consumption. Thus, cardiac ANS activity is a major determinant of circadian HR variations in mice independent of physical activity. Physical activity can strongly amplify the daily HR fluctuations in mice when given free access to running wheels.

## Data Availability Statement

The raw data supporting the conclusions of this article will be made available by the authors, without undue reservation.

## Ethics Statement

The animal study was reviewed and approved by the York University Animal Care Committee (ACC).

## Author Contributions

Experiments were performed at the York University, Department of Biology. NB, RL, and PHB were responsible for the conception, the design, the acquisition, analysis, and interpretation for the work, drafting the work, and revising it critically for important intellectual content. NP was involved in acquisition, analysis, and interpretation of data, and drafting the work. RD and SY were involved in acquisition, analysis, and interpretation of data for the work. All authors approved the final version of this manuscript and agreed to be accountable for all aspects of the work.

## Funding

This work was supported by a Project grant (PJT-125950) and a Canada Research Chair in Cardiovascular Biology from the Canadian Institutes of Health Research to PHB. Support was also provided from the Canadian Foundation for Innovation, John Evans Leader Award to PHB. Canadian Institutes of Health Research (CIHR) Fellowship to RL.

## Conflict of Interest

The authors declare that the research was conducted in the absence of any commercial or financial relationships that could be construed as a potential conflict of interest.

## Publisher’s Note

All claims expressed in this article are solely those of the authors and do not necessarily represent those of their affiliated organizations, or those of the publisher, the editors and the reviewers. Any product that may be evaluated in this article, or claim that may be made by its manufacturer, is not guaranteed or endorsed by the publisher.
