# Supplementary material for: Dissecting the Roles of the Autonomic Nervous System and Physical Activity on Circadian Heart Rate Fluctuations in Mice
Source: Front Physiol. 2021 Oct 18;12:692247. doi: 10.3389/fphys.2021.692247 (PMC8558381; doi:10.3389/fphys.2021.692247)
Supplement: Supplementary file 1 [file Data_Sheet_1.docx]

Supplementary Material

# Supplementary Materials and Methods

**1.1 Experimental Animals**

All experiments were conducted on 10-week old adult male CD1 mice (Charles River Laboratories, Wilmington, MA, USA) with a body weight (BW) range of 32-39 g. All mice were housed in temperature-controlled rooms (22°C) with 12:12-hour light:dark cycles in the vivarium at York University and give ad libitum access to food and water. Mice were given 1 week to acclimate following their arrival to the animal facility before performing an experiments. All Experimental protocols were in compliance with the standards of the Canadian Council on Animal Care.

**1.2 Heart Rate Measurements and lighting conditions for anesthetized mice**

Diurnal fluctuations in HR were measured in anesthetized using surface electrocardiogram (sECG) recordings at several time points (i.e. zeitgeber times (ZT)). ZT 0 refers to the time when lights are turned on while ZT 12 refers to when lights are turned off. ZT 6 refers to the time when the lights have been on for 6 hours and ZT 18 refers to the time when the lights have been off for 6 hours.

Mice were anesthesized using isoflurane in 100% oxygen administered via a Fluotec Mark 2 Vaporizer (Cyprane, Keighley, UK) with the percent isofluorane (typically ~1.5%) adjusted to maintain breathing rates between 90-110 breaths per minute (Lakin et al., 2018). Core temperature was continuously monitored using a rectal temperature probe (THM 150, Indus Instruments, Webster, TX, U.S.A) and maintained between the physiologically relevant parameters of 36.9−37.3°C. Surface electrocardiograms (sECGs) were recorded using platinum sub-dermal electrodes (F-E7, Grass Technologies, West Warwick, RI, USA) in the lead II configuration, connected to a Gould ACQ-7700 Amplifier controlled by the Ponemah software (Data Sciences International, New Brighton, MN, USA). A minimum equilibration period of 15 min was used before recording baseline heart rate for 20 minutes. Three sECG recordings were made for each mouse in each time point and each measurement required 1.5-2 hours to complete.

Heart rates were calculated from R-R intervals. When making sECG measurements in anesthetized mice during the dark periods, we minimized light exposure in order to minimize disruption of the circadian rhythm by performing the sECG measurements in rooms equipped with LED lights (< 1 lux) emitting red light (620-750 nm) which is outside the range of wavelengths detected by murine retinas (Hattar et al., 2003).

To create a complete profile of circadian fluctuations, mice housed in either standard light-dark (LD) rooms in which case ZT 0 corresponds to 7_AM_ or “reverse” dark-light (DL) rooms in which case ZT 0 corresponds to 7:30_PM_. This allowed estimation of HRs at ZT0, ZT 6 and ZT 12 using mice housed in the LD rooms and HRs at ZT 12, ZT 18 and ZT 24/0 using mice housed in the DL rooms. For all time points, HRs were estimated on 3 separate occasions (triplicates) for every mouse, with the repeated measurements always separated by at least 2 days.

Studies were performed in two groups of mice. In the first group (n=7), mice were housed in rooms either with LD lighting (n=4) thereby allowing the HRs to be estimated at ZT0, ZT6 and ZT12 or with DL lighting (n=3) thus allowing HRs to be estimated at ZT12, ZT18 and ZT24/0. HRs were estimated in triplicate with a minimum of 2 days between measurements. The mice in the LD rooms were then switched to DL rooms while the mice in DL rooms were placed in LD rooms. After 10 days to allow to acclimatize to the new light conditions, mice HRs were again estimated at ZT0, ZT6 and ZT12 for the mice (n=3) in the LD rooms and at ZT12, ZT18 and ZT24/0 for the mice (n=4) in DL room. HR was again measured in triplicate. In the second group (n=6), mice were housed in rooms with LD lighting (n=3) or in DL rooms (n=3). HRs were estimated at the appropriate times in triplicate. Mice were not switched to different rooms thereafter. Detailed subgroup comparisons revealed no differences between the daily HR variations in the 2 groups of mice. Consequently, the HR data from all 13 mice were pooled for all the analyses presented.

All sECG recordings were made before and after administration of autonomic blockers. Autonomic blockers were introduced in two different sequences: (1) intraperitoneal (IP) injection of propranolol (10 mg/kg BW) to block cardiac sympathetic nerve activity (SNA) followed by IP atropine injection to achieve total autonomic blockade or (2) IP atropine injection to block parasympathetic nerve activity (PNA) followed by propranolol IP treatment to (again) achieve total autonomic blockade. HRs were estimated from the sECG recordings 25 minutes after the administration of autonomic blockers.

**1.3 Heart rate recordings in Conscious Mice**

HR assessments in conscious animals were made by surgically implanting radio telemetry units, as described below. Following the surgery, mice (n=5) were placed in individual cages, with ad libitum bedding, food, and water supply, housed under a reverse 12-h:12-h DL cycle and were allowed 7 days of recovery prior to recording. Thereafter, ~ 60-hour recordings were collected over the weekend for 2 weeks. Following the first weekend recording (week 1), mice were given access to running wheels installed in their individual cages (for week 2). Following the introduction of running wheels, we collected HR data for only 1 week to isolate the effects of physical activity on circadian fluctuations in HR from any cofounding factors, mainly potential exercise-induced autonomic remodeling.

Radiofrequency emitting ECG devices (EA-F20, Data Sciences International, New Brighton, MN, USA) were surgically implanted into 8 week-old male CD1 mice (Charles River Laboratories, Wilmington, MA, USA). All surgeries were conducted under sterile conditions. Animals were anesthetized (~2.5% isoflurane induction, 1.5-2% isoflurane maintenance), administered Metacam (2mg/kg, s.c.) and placed on a heating platform to maintain core temperature at 37⁰C. The ventral abdomen was shaved, disinfected using disinfectant soap and rinsed with water followed by 95% alcohol/betadine. Subsequently, a midline incision was made in the skin in the upper abdomen and a second incision was made in the junction of the right shoulder with the neck. The telemetric device was placed subcutaneously on the lateral abdomen using the first incision. The electrodes were passed under the skin to either the right shoulder/neck (negative lead) and to the left lateral abdomen (positive lead). Both leads were tethered to the underlying musculature using 6-0 silk suture. Thereafter, both incisions were flooded with saline and closed with 6-0 silk suture.

All recordings were collected continuously at a 1000Hz sampling rate. Data was acquired and analyzed offline using Ponemah P3 Plus software (v6.4, Data Sciences International, New Brighton, MN, USA).Heart rates were determined from RR-intervals using Ponemah P3 Plus software.

**1.4 Respirometry measurements in Conscious Mice**

Wild-type CD1 mice were housed individually in custom-built metabolic cages with access to water and standard mouse chow ad libitum. Humidified (~40% H_2_O) air was continuously injected into the metabolic cages at 1L/min. Simultaneous tracking of running parameters (running speed, running cumulative distance) was performed using custom-built running wheels, atmospheric parameters (air temperature, atmospheric pressure and %H_2_O) using BME-280 sensors (Adafruit, New York City, USA). Outgoing cage gas (%O_2_ and %CO_2_) was desiccated and sampled using an iWORX GA-200 O_2_/CO_2_ gas analyzer (iWORX Dover, USA) at 0.5L/min. V_O2_ consumption rates were calculated, using a standard respirometry equation:

V_O2_ = Δ%O_2_ * (P_atm_– P_H2O_) * F

where Δ%O_2_ = difference in %O_2_ between incoming and outgoing air flowing, F is the rate of air flow into the cage, P_atm_ = atmospheric pressure, and P_H2O_ = partial pressure of H_2_O = f(%humidity and temperature).

**1.5 Statistical Analysis**

HR data results are reported as mean ± standard deviation (SD). To detect circadian HR fluctuations, the pooled HR data for all the mice, at different time points, were fit nonlinearly using the Least Squares (ordinary) Fit algorithm, provided through the program GraphPad Prism (GraphPad Software, Inc) to the following sine function:

Y = A * sin ((2 * π * T / 24) + φ) + B

where A is the amplitude (i.e. one half the peak to trough variation) expressed in heart beats per minute, φ is the acrophase (i.e. the time delay from ZT0 of the HR cycle) expressed in radians, B is the baseline offset which equals the mean heart rate over 24 hours (i.e. the MESOR) expressed in heart beats per minute, T is the time of day in hours, π = Pi radians = 3.14159 radians. Fits were constrained to a 24-hour wavelength given the controlled 12:12 light:dark cycle. Zero amplitude (no rhythm) tests were used to determine the presence of a circadian rhythm in HR. Differences in fit parameters were determined within and between groups using an extra sum-of-squares F test. To assess the robustness of our approach, the presence of circadian HR fluctuations was also assessed using nonlinear fits the HR results for each individual mouse to a sine function (**Supplemental Figure 1**). In this case, the nonlinear fitting routine provided an estimate of “A” and “φ “ for each mouse which were then used to determine whether “A” (mean ± SD) was non-zero using a T-Test (*P* < 0.05). A two-way mixed model repeated measures ANOVA with Holm- Šidak correction for multiple comparisons was used to compare circadian HR fluctuations between groups of anesthetized (i.e. with and without pharmacological blockade) or conscious mice (i.e. with or without free wheel access). An independent (two-tailed) student’s t-test was used to assess differences between anesthetized and conscious mice. *P* values < 0.05 were considered significant.

# Supplementary Figures and Tables

## Supplementary Figure Captions

**Supplementary Figure 1. Variation in surface ECG recorded mean HR collated from individual nonlinear fits of anesthetized mice throughout the day. A)** The left panel shows HR results of anesthetized mice (n=13) under baseline conditions along with the sine function fit using the mean amplitude (middle panel) and mean phase (right panel). The amplitude (middle panel) and phase (right panel) results were obtained by performing nonlinear fits the HR data for each individual mouse to a sine function (with a 24-hour period). The amplitude was determined to be greater (*P*<0.0001) than zero. **B**) The results (n=7) as described in (**A**) but the HR data was obtained after administration of propranolol (10 mg/kg BW). The amplitude was greater (*P*=0.0001). **(C)** The results (n=6) as described in (**A**) but the HR data was obtained after administration of atropine (2 mg/kg BW). The amplitude was greater ($P=0.004) than zero. **D)** The results (n=6) as described in A) but the HR data was obtained after administration of both propranolol and atropine. The amplitude was greater (P=0.03) than zero. **E)** The left panel shows telemetry HR data for conscious mice. The line shows the curve predicted the nonlinear sine function fits to the HR data. The predicted non-zero (*P*<0.0001) amplitude shown in the middle panel is derived from individual fits to each mouse. **F)** The same data as outlined in **E)** but for conscious mice after having access to running wheels for 1 week. Again, the nonlinear individual fits yield a non-zero (*P*<0.0001) amplitude shown in the middle panel. Data is presented as Mean±S.D. &*P*<0.0001 and n.s, (*P*=0.24 for C and *P*=0.31 for D) using a two-way mixed-model repeated measures ANOVA (left panel) **P*<0.05, ***P*<0.01, ****P*<0.001; $*P*<0.0001 using a two-tailed Student’s T-Test based on the zero amplitude (no rhythm) test (middle panel).

**Supplementary Figure 2**. **Typical oxygen (O_2_) measurements presented as O_2_ consumption rates (V_O2_) in mice before and 1 week after gaining access to running wheel.** **A, B**) Sample 24-hour V_O2_ measurements in a wild-type CD1 mouse just prior to **(A)**, and 1 week after **(B)** gaining access to a running wheel. The measurements in the dark and light phase are indicated. **C, D)** Frequency distribution of V_O2_ values for the sample recordings shown in panels A and B. Notice that the distribution of V_O2_ values shows two peaks in the absence of a running wheel (**C**) while after a running wheel is introduced the V_O2_ histogram shows 3 peaks (**D**). The V_O2_ values associated with the two lower peaks in mice with access to wheels is align closely with the two V_O2_ peaks seen in the absence of the wheel. The levels of the thresholds used to distinguish between the different V_O2_ levels are shown as lines in panels **A** and **B**. Also shown in panel **B** is the distance run by the mouse. Notice that mice only engage in running during the dark phase.

## Supplementary Figures

**Supplementary Figure 1**


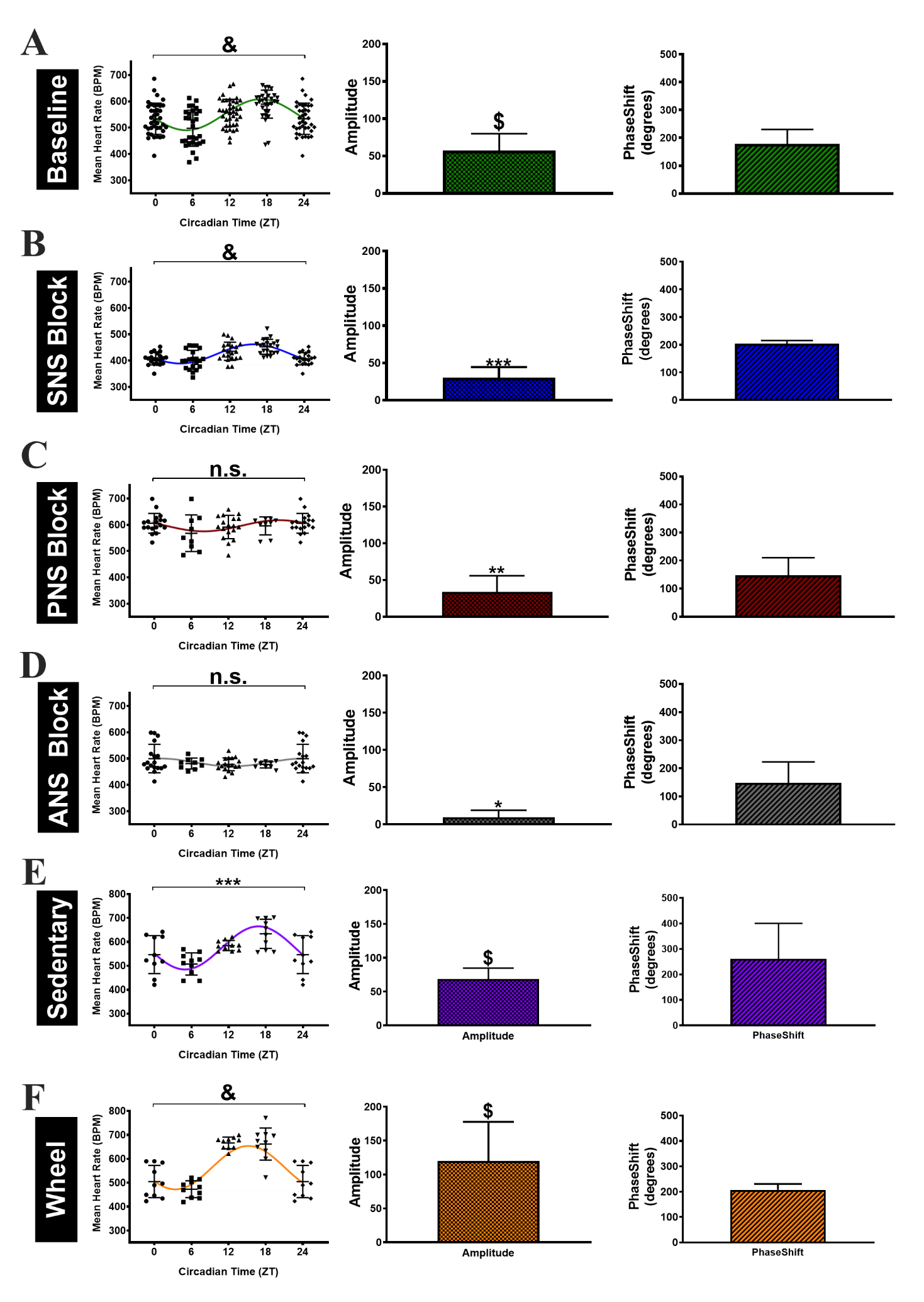


**Supplementary Figure 2**

**2.2 Supplementary Tables**

**Supplementary Table 1. Summary of Amplitudes and Phases Estimated from Individual Non-linear Sine Function Fits to the HR Measurements Under the Indicated Experimental Conditions**

| **Condition** | ***N*** | **Absolute HR** | | | **Normalized HR** | |
| --- | --- | --- | --- | --- | --- | --- |
|  |  | **Mean HR**  **(BPM)** | **Amplitude** | **PhaseShift (radians)** | **Amplitude** | **PhaseShift (radians)** |
| **Anesthetized Mice** | | | | | | |
| **Baseline** | **13** | **544±65** | **57.13 ±22.8** | **2.91±0.12** | **0.12±0.05** | **3.11±0.9** |
| **SNA**  **Block** | **7** | **424±36** | **30.1±14.29 ^n.s.^** | **2.59±0.14** | **0.08±0.03 ^n.s.^** | **3.56±0.2** |
| **PNA**  **Block** | **6** | **592±46** | **33.99±21.9 ^n.s.^** | **2.59±0.46** | **0.04±0.03^*.^** | **2.57±1.1** |
| **ANS**  **Block** | **6** | **485±36** | **9.72±9.5^**^** | **1.41±0.79** | **0.04±0.04^*^** | **2.58±1.2** |
| **Conscious Mice** | | | | | | |
| **SED** | **5** | **568±53** | **68.9±17.8 ^n.s.^** | **4.55±2.43** | **0.12±0.03 ^n.s.^** | **4.55±2.4** |
| **WHEEL** | **5** | **568±53** | **119.89±57.7^***, $^** | **3.61±0.40** | **0.18±0.10 ^n.s.^** | **3.61±0.40** |

The amplitudes and phase shifts were obtained by performing nonlinear fits the HR data for each individual mouse to a sine function (with a 24-hour period) under various conditions: Baseline = no blockers, SNA Block = with propranolol, PNA Block = with atropine, ANS Block = with atropine and propranolol, SED = conscious mice with no wheel access, WHEEL = conscious mice with wheel access. Data presented as Mean±S.D.***P*<0.01, ****P*<0.001, and n.s. (*P*=0.22 for SNA Block; *P*=0.35 for PNA Block; *P*=0.64 for SED) compared to Baseline using an two-way repeated measures ANOVA with Holm-Šidak multiple comparison test. $*P*<0.05 and and n.s (*P*=0.46 for SNA Block; *P*>0.99 for SED; *P*=0.22 for WHEEL) compared to conscious sedentary mice with no wheel access (SED) using a two-way repeated measures ANOVA with Holm-Šidak multiple comparison test.

**3. References**

Hattar, S., Lucas, R.J., Mrosovsky, N., Thompson, S., Douglas, R.H., Hankins, M.W., Lem, J., Biel, M., Hofmann, F., Foster, R.G., and Yau, K.W. (2003). Melanopsin and rod-cone photoreceptive systems account for all major accessory visual functions in mice. *Nature* 424**,** 76-81.

Lakin, R., Guzman, C., Izaddoustdar, F., Polidovitch, N., Goodman, J.M., and Backx, P.H. (2018). Changes in Heart Rate and Its Regulation by the Autonomic Nervous System Do Not Differ Between Forced and Voluntary Exercise in Mice. *Front Physiol* 9**,** 841.
